# Portomesenteric venous thrombosis in a prophylactically anticoagulated obese patient after laparoscopic sleeve gastrectomy: a case report

**DOI:** 10.1186/s13256-021-03174-w

**Published:** 2021-12-17

**Authors:** Feras Alsannaa, Faisal Albaqami, Mishary Shalhoub

**Affiliations:** 1 Prince Sultan Military Medical City, Riyadh, Saudi Arabia; 2 King Abdullah bin Abdulaziz University Hospital, Riyadh, Saudi Arabia

**Keywords:** Bariatric surgery, Embolism, Intestine, small, Ischemia

## Abstract

**Background:**

Obesity is associated with an increased risk of morbidity and mortality, so weight reduction is important. Bariatric surgery is a well-tolerated approach for reducing body weight, with laparoscopic sleeve gastrectomy commonly performed. An uncommon and potentially fatal sequela of laparoscopic sleeve gastrectomy is portomesenteric vein thrombosis, which may result in severe bowel ischemia.

**Case report:**

A 32-year-old Middle Eastern obese man (body mass index 33) presented to the emergency department with severe, generalized abdominal pain 2 weeks after laparoscopic sleeve gastrectomy. Computed tomography of the abdomen and pelvis revealed extensive acute on chronic portosplenic and superior mesenteric vein thrombosis with associated small bowel ischemia. Laparoscopic exploration was converted to midline laparotomy and an extensive ischemic small bowel resection.

**Conclusion:**

Laparoscopic sleeve gastrectomy carries a risk of both morbidity and mortality. Venous thromboembolism is a well-known risk of bariatric surgery, but portomesenteric vein thrombosis is also a rare but sometimes serious complication. A high index of suspicion for portomesenteric vein thrombosis to prompt early detection is essential in patients who have undergone laparoscopic sleeve gastrectomy to minimize complications and optimize outcomes. Uncertainty still remains around the optimal dose and duration of anticoagulation after laparoscopic sleeve gastrectomy.

## Background

Obesity is an accepted risk factor for increased morbidity and mortality [1], and the life expectancy of morbidly obese individuals is reduced by ~5–20 years [2]. When patients lose weight, it increases their quality of life and reduces mortality rates [3]. Surgery is a well-tolerated modality for reducing body weight. Laparoscopic sleeve gastrectomy (LSG) was originally considered a bridge to definitive Roux-en-Y gastric bypass in high-risk patients [4]. However, LSG was shown to have a positive effect on weight reduction not only through gastric restriction but also through appetite suppression [5–7], so it became a widely performed and standard weight reduction procedure for most bariatric surgeons. Morbidly obese patients are at high risk of venous thromboembolism (VTE), and one uncommon but potentially fatal sequela of LSG is portomesenteric vein thrombosis (PMVT), which can cause severe bowel ischemia [8, 9]. Here we report a rare case of bowel ischemia secondary to PMVT in an LSG patient receiving prophylactic anticoagulation.

## Case report

An obese 32-year-old Middle Eastern man with a body mass index (BMI) of 33 presented to the emergency department (ED) with a 6-day history of severe, worsening, generalized abdominal pain and vomiting. He had undergone LSG 13 days prior to the current admission at a private hospital. He was prescribed a protein pump inhibitor and enoxaparin 40 mg daily, which he had taken regularly.

On examination, the patient looked unwell, dehydrated, and in pain. His Glasgow Coma Scale score was 15, and he had a heart rate of 135 beats per minute, blood pressure 132/82 mmHg, respiratory rate 20 breaths per minute, and body temperature 36.5 °C. On examination, his abdomen was distended with generalized tenderness, but his laparoscopic wounds had healed.

**Fig. 1:**
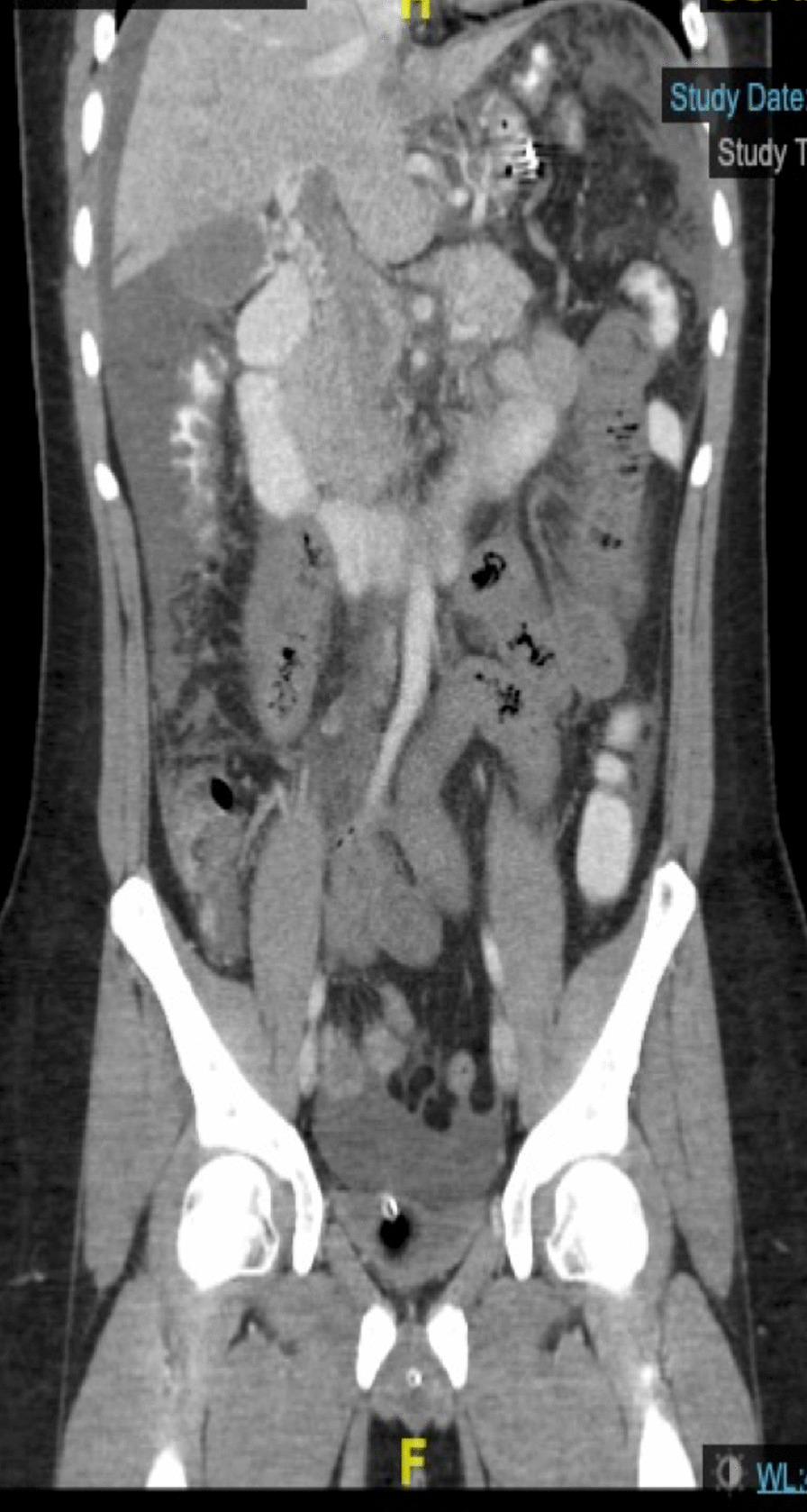
CT abdomen and pelvis demonstrating PVMT and signs of small bowel ischemia

Initial laboratory investigations revealed a white blood cell count (WBC) of 27,300/μL (4000–11,000 μL), hemoglobin 17.3 g/dL (10–15 g/dL), and serum lactate 7.6 mmol/L (0.5–1.9 mmol/L). Computed tomography (CT) of the abdomen and pelvis with intravenous contrast revealed extensive acute on chronic portosplenic and superior mesenteric vein thrombosis, with consequent small bowel ischemia (Fig. 1).

**Fig. 2:**
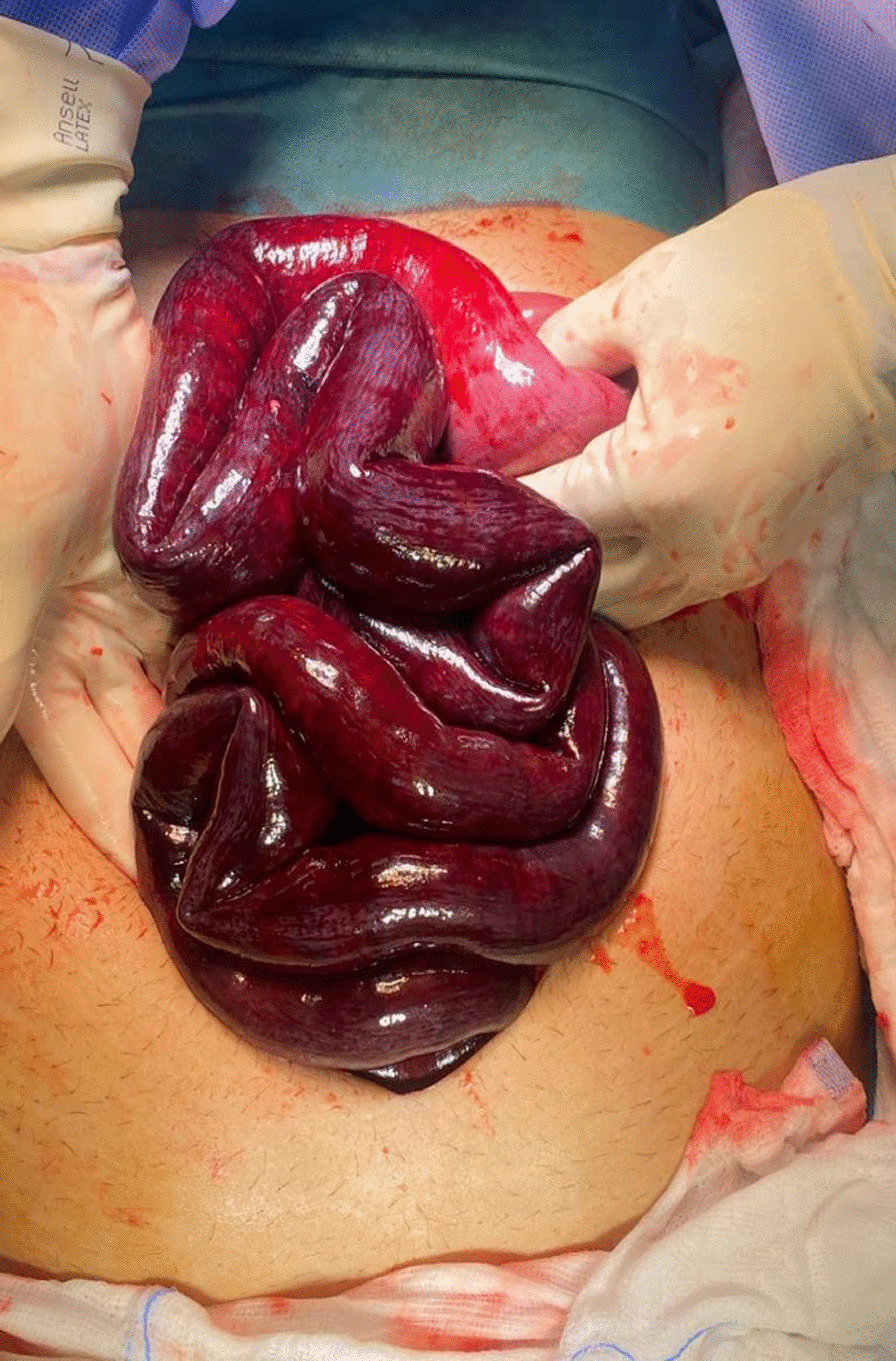
Abdomen with small bowel ischemia

**Fig. 3:**
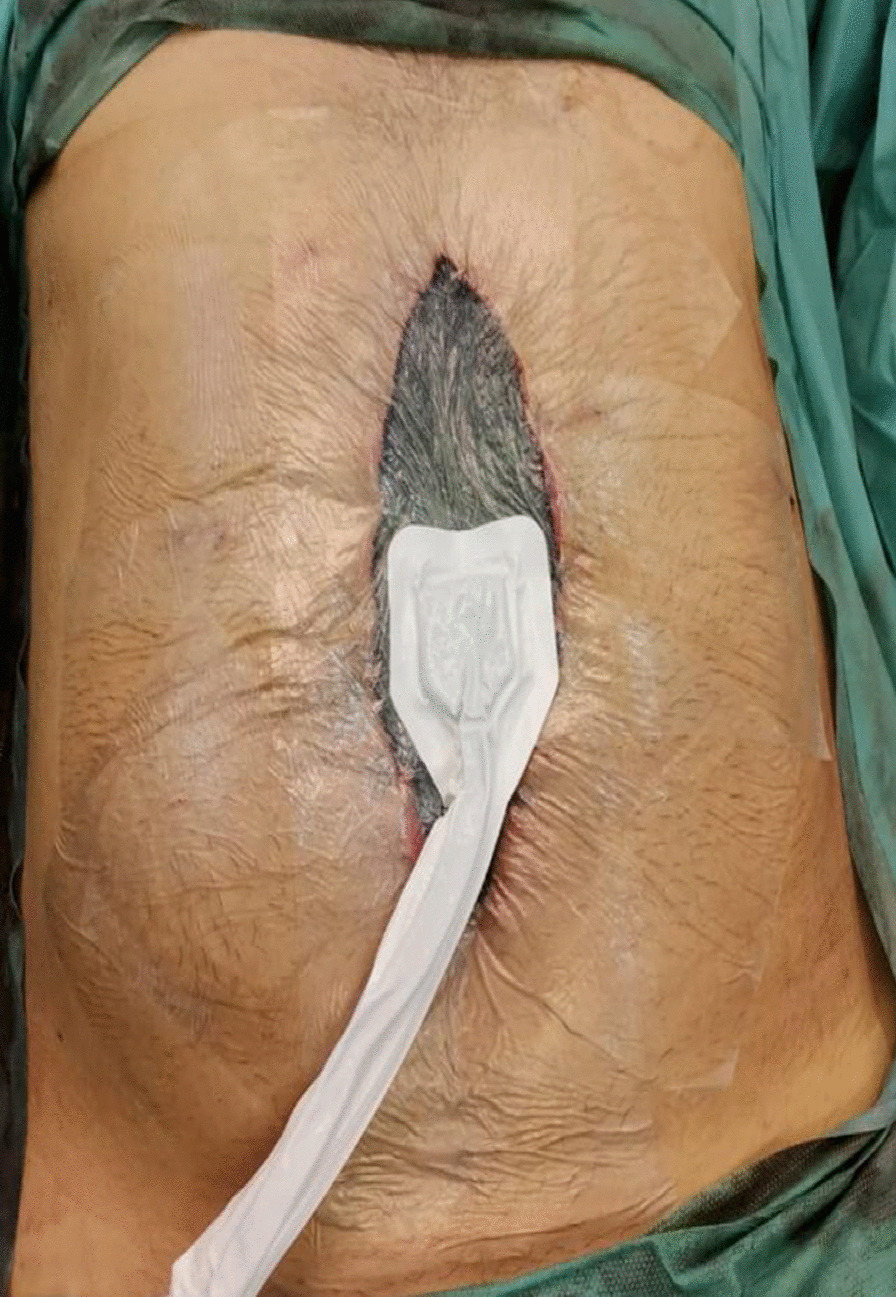
Temporary abdominal closure with a vacuum-assisted closure device

He was admitted under the care of the acute surgical team and resuscitated, and underwent laparoscopic exploration, which confirmed the CT findings. After conversion to midline laparotomy, 255 cm of small bowel was resected (Fig. 2) and the abdomen was left open with a vacuum-assisted closure device dressing (Fig. 3). The patient was transferred to the intensive care unit. A second look was carried out 24 hours later, and both the small and large intestines appeared healthy, so primary anastomosis and abdominal closure were performed. A solid diet was introduced gradually, and the patient was discharged home on day 12 postoperation on warfarin. A thrombophilia screen was negative. The patient was seen multiple times for follow-up; he was tolerating oral intake and had reduced his BMI to 19 with no clinical manifestations of short bowel syndrome.

## Discussion

In the current era of weight-reducing surgery, LSG is one of the most popular operations performed by bariatric surgeons. LSG not only has an excellent outcome in terms of weight reduction and remission of obesity-related comorbidities, but it is also associated with lower morbidity due to its technical simplicity [10]. However, LSG still has a risk of morbidity (17.5%) and even death (0–1.2%) [11].

One of the well-known complications of bariatric surgery is VTE, which can occur due to both local and systematic factors [12]. The risk of VTE can be classified as either patient-related or surgery-related. A high BMI alone is considered an independent risk factor for the development of VTE [13]. Finks *et al*. showed that every 10-unit increment in BMI was associated with a 37% increase in VTE risk [14]. Other risk factors include male gender, a smoking history, and longer operative time. PMVT is rare complication of LSG but has been reported in up to 1% of cases [15]. The literature indicates that PMVT tends to occur more frequently in LSG than other types of bariatric surgery, which might be explained by thermal injury to blood vessels near the greater curvature, splenic infarction, liver congestion and stasis, or poor oral intake with dehydration [16].

Based on this risk of developing VTE after bariatric surgery and in obese individuals, extended prophylactic anticoagulation is indicated, with both low molecular weight heparin and unfractionated heparin generally used. Although there is no level I evidence regarding the dose and duration of anticoagulation, some studies have shown that a prolonged postoperative course may be beneficial for decreasing the risk of VTE, while preoperative prophylactic anticoagulation is associated with a high risk of bleeding without decreasing VTE risk; therefore, it is not recommended. Most postsurgery VTE occurs within 30 days of discharge [17]. In our case, although the patient was compliant with his prophylactic anticoagulant, he developed PMVT, highlighting this uncertainty around the optimal dose and duration of anticoagulation.

The management of PMVT is mainly dictated by the chronicity of clot formation, that is, whether it is acute or chronic, and the sequelae of the thrombosis. Conservative management is appropriate in many cases, but prognosis and outcome depend on the time of presentation and the development and nature of any complications. A surgical approach is reserved for those patients who have developed signs of intestinal ischemia [18]. The prognosis of PMVT ischemia is much better than for other forms of mesenteric ischemia (for example, arterial and non-occlusive), primarily due to early discovery and intervention. Our patient presented to the ED after 6 days of symptoms, which led to the resection of a large segment of ischemic small bowel and placing him at risk of developing short bowel syndrome. After stabilization of the patient, therapeutic anticoagulation is usually extended for at least 3–6 months if no thrombophilia is identified [19]. Back to our case, CT showed acute on chronic portosplenic and superior mesenteric vein thrombosis, which raised the question about preoperative need for assessing portal system to prevent such major complication.

## Conclusions

LSG is a commonly performed bariatric surgery that is technically simple but nevertheless carries a risk of morbidity and mortality. PMVT should be considered with a high index of suspicion in the differential diagnosis in any patient post LSG with signs of peritonitis. Further studies are needed to determine the adequate duration and dosing of prophylactic anticoagulation post LSG and application of portal venous system study as part of perioperative screening.

## Abbreviations

LSG: Laparoscopic sleeve gastrectomy
PMVT: Portomesenteric vein thrombosis
VTE: Venous thromboembolism
ED: Emergency department
